# Staff-to-resident abuse in nursing homes: a scoping review

**DOI:** 10.1186/s12877-022-03243-9

**Published:** 2022-07-06

**Authors:** Julian Hirt, Laura Adlbrecht, Steffen Heinrich, Adelheid Zeller

**Affiliations:** 1 Center for Dementia Care, Institute of Applied Nursing Sciences, Department of Health, Eastern Switzerland University of Applied Sciences, Rosenbergstrasse 59, 9000 St.Gallen, Switzerland; 2 International Graduate Academy, Medical Faculty, Institute for Health and Nursing Science, Martin Luther University Halle-Wittenberg, Magdeburger Strasse 8, 06112 Halle (Saale), Germany; 3 Department of Clinical Research, University Hospital Basel, University of Basel, Basel, Switzerland

**Keywords:** Elder abuse, Physical abuse, Aggression, Nursing home, Long-term care

## Abstract

**Background:**

Elder abuse in long-term care is an important public health concern with social,

health-related, and economic implications. Staff-to-resident abuse is of particular interest since institutions should protect residents’ rights and prevent harm. To provide an up-to date comprehensive overview of staff-to-resident abuse in nursing homes, we performed a scoping review considering types of abuse, their prevalence and associated factors, descriptions, experiences, and preventive interventions.

**Methods:**

We performed a scoping review following the framework provided by Arksey and O’Malley. We searched MEDLINE (via PubMed), CINAHL, PsycINFO via Ovid, and Cochrane Library.

Additionally, we performed free web searching using Google Scholar and checked relevant reviews. Two reviewers independently selected studies. We narratively synthesised the results.

**Results:**

Out of 3876 references retrieved by our search, we included 46 studies in 47 reports. The prevalence rates of abuse varied widely, ranging from 0 to 93% depending on the type of abuse. Associated factors of abuse at the staff, resident, and nursing home level were evaluated inconsistently. Abuse was perceived ambiguous: even though it was considered unacceptable, it was underreported. We found only four studies addressing preventive interventions. Of these, four made recommendations for intervention development. Only one study with an experimental design examined a multi-component intervention including education and mutual support.

**Conclusions:**

The review yielded heterogenous evidence not allowing a concrete conclusion on prevalence and associated factors. However, the results show the significance of the problem and indicate that there are associate factors of abuse that can be influenced by appropriate interventions. These are amongst other staff education, organisational culture, and conditions. Further research should investigate the composition and content of preventive interventions and their potential to reduce abusive behaviours.

**Supplementary Information:**

The online version contains supplementary material available at 10.1186/s12877-022-03243-9.

## Background

Elder abuse in long-term care is an important public health concern with social, health-related, and economic implications. The World Health Organization defines elder abuse as a single or recurrent act resulting in harm or distress of an older person in a trust relationship [1]. Classifications of abuse comprise emotional or psychological abuse (verbal or non-verbal behaviour, e.g., humiliation, threats, harrassment, isolation), physical abuse (intentional physical force resulting in physical harm or distress, e.g., hitting, pushing, inappropriate use of medication, force-feeding), sexual abuse (forced or unwanted sexual interaction with touching and non-touching acts), financial abuse (unauthorized use of resources, including fraud and theft), and neglect (caregivers’ failure to meet the person’s essential needs, e.g., nutrition, hygiene, medical care) [2]. In a current meta-analysis, resident-reported prevalence of abuse was highest for psychological abuse (33.4%), and lowest for sexual abuse (1.9%) [3]. Consequences of abuse for residents are reduced quality of live, psychological and physical symptoms, increased morbidity and premature mortality [4].

Parties involved in abuse in nursing homes are residents, relatives, and staff members [5]. Staff-to-resident abuse is of particular interest since institutions should protect residents’ rights and prevent harm [6]. In our manuscript, we use the term staff for all members of the nursing team indiscriminate of their qualification level (e.g., registered nurses and nursing assistants). The risk of staff-to-resident abuse is a multifactorial problem influenced by resident-related, staff-related and organization-related characteristics [7–9]. Mobility limitations and increased need for assistance with activities of daily living are strongly associated with the risk of being abused [10]. People with dementia (in many cases representing the majority of residents) are particularly vulnerable due to their reduced cognitive capacity to recognise and report abuse [11].

Staff-resident relationships in nursing homes are characterized by imbalances of power and residentsʼ dependency on staff to satisfy their needs [12, 13]. In addition, nursing competence in long-term care is often not adequate and may entail abuse [14]. Furthermore, nurses feel stressed and report not having completed at least one task in the last service due to scarcity of resources and abundance of services to be provided [15, 16]. They often feel powerless, overwhelmed and restricted in their freedom of action due to prevailing conditions [17]. Furthermore, institutional regulations and resource planning strategies can be abusive as such if they affect residents’ self-determination in everyday activities [18].

Studies on staff-to-resident abuse require that participants (residents, staff members or other persons) recognize abuse, recall past events, consider them worth reporting, and avoid socially desirable responses [19, 20]. The challenges of assessing staff-to-resident abuse are met by taking into account different perspectives, interviewing various people involved, and using specific methods, assessment tools, and questioning techniques. As a result, there is a large body of evidence reporting prevalence, conceptualizations, associated factors, and interventions addressing staff-to-resident abuse.

Literature reviews have been conducted for subgroups (e.g., residents with dementia) [11, 21] or specific types of abuse (e.g., sexual abuse) [22]. Some reviews have been published years ago [23, 24] and, therefore, do not consider recent studies investigating staff-to-resident abuse in nursing homes. To provide an up-to-date comprehensive overview of staff-to-resident abuse in nursing homes, we aimed to answer the following three questions:

- (1) How often does staff-to-resident abuse occur in nursing homes (per type of abuse)? What are associated factors of staff-to-resident abuse in nursing homes?
- (2) How is staff-to-resident abuse described and experienced in nursing homes?
- (3) Which interventions are aimed at preventing staff-to-resident abuse in nursing homes?

## Methods

We conducted a scoping review following the framework provided by Arksey and O’Malley [25]. To structure our study report, we used the “Preferred Reporting Items for Systematic reviews and Meta-Analyses extension for Scoping Reviews” (PRISMA-ScR) [26]. We depicted the literature retrieval and selection process by means of the PRISMA 2020 flow diagram and PRISMA-S [27, 28]. We did not register the review.

### Eligibility criteria

We included quantitative, qualitative, and mixed-methods studies following the IMRad format (“Introduction, Methods, Results, and Discussion”). Studies in English and German addressing staff-to-resident abuse in nursing homes had to be published in academic journals since the year 2000.

In case of mixed samples from nursing homes and other settings, 80% of the population had to be related to nursing homes. We define nursing homes as a setting where people (can) receive 24­hour professional nursing [29]. We excluded studies with an unclear or smaller amount of nursing home-related populations. In addition, we did not include reviews, commentaries, opinion papers, policy statements, and study protocols as well as studies published as books, theses, reports, conference proceedings, and abstract-only publications. We also did not consider studies addressing self-directed violence (e.g., self-aggression or self-neglect), resident-to-resident abuse or resident-to-staff abuse. Additionally, we did not take into account studies conducted in acute care, home care, day care, specialised psychiatric care, assisted living facilities, and institutions for people with physical and/or intellectual disabilities.

### Information sources

To address our study objectives, we searched MEDLINE (via PubMed), CINAHL, PsycINFO via Ovid, and Cochrane Library in June 2020. As supplementary search methods, we performed free web searching using Google Scholar. Additionally, we checked relevant reviews reporting a systematic search following the IMRaD structure. We retrieved these reviews while searching for further primary studies on staff-to-resident abuse.

### Search

Two experienced reviewers developed the search strategy. To address all three review questions, the search strings contained two search components, namely synonyms and descriptions of abuse (such as violence, mistreatment, and aggression), and synonyms and descriptions of nursing home (such as long-term care and care home). We identified free text search terms based on preliminary topical searches, brainstorming within our research group, and by using a thesaurus [30]. Database-specific controlled vocabulary complemented our search terms. For free text terms, we used wildcards and/or quotation marks to develop the final search strings. To specify our search approach, we restricted our free text terms to the title and abstract field. We searched controlled vocabulary in the corresponding search fields as well as in the title and abstract field. The final search strings for all databases are part of supplementary material S1. For our free web search, we used the same search terms iteratively entered in Google Scholar.

### Selection of sources of evidence

Following recent guidance to minimise the risk of missing relevant studies, two reviewers independently screened titles, abstracts, and full texts against eligibility criteria [31]. To manage independent study selection, we used an web-based screening tool, Rayyan [32]. Disagreements were discussed between the two reviewers until consensus was reached.

### Data charting process

Two reviewers developed a standardised data extraction table based on preliminary extractions of three included studies. One reviewer extracted data, thereby using full study reports. A second reviewer peer-checked extracted data. We did not request any additional data from the authors.

### Data items

For all studies, we extracted bibliographic information and data concerning country, study aims, design, participants, sample size, sampling strategy, type of abuse as well as methods of data collection and analysis.

To answer our first review question, we additionally extracted reference time (retrospective/prospective), characteristics of residents (e.g., gender, age), characteristics of staff (e.g., gender, age, professional experience), “observed prevalence” per type of abuse (i.e., staff-to-resident abuse observed or witnessed by staff), “committed prevalence” per type of abuse (i.e., staff-to-resident abuse committed and self-reported by staff), “reported prevalence” per type of abuse (i.e., staff-to-resident abuse experienced abuse or observed abuse, reported by residents and/or others), resident-related factors (e.g., gender, disease, age), staff-related factors (e.g., gender, age, education, experience), as well as institution-related factors (e.g., country, region).

To answer our second review question, we additionally extracted the descriptions and experiences provided by study authors as a verbatim quote.

To answer our third review question, we additionally extracted information concerning recipients, facilitators, and characteristics of the intervention, control interventions, outcomes, and main results. Regarding the main results of qualitative studies to answer this research question, we summarized preventive interventions addressing staff-to-resident abuse and extracted quotes.

### Synthesis of results

One reviewer narratively synthesised data for extracted items for each of our research questions based on tabular data extraction. To answer our first review question on prevalence and associated factors of staff-to-resident abuse, we clustered types of abuse using the classification provided above: psychological (including emotional, mental, and verbal abuse), physical, sexual, financial (including material abuse fraud and theft), or other abuse (including care mistreatment/maltreatment, rights violation, discriminatory abuse, medication abuse, e.g., delayed or denied access to medications, and neglect [including mental and physical neglect]) [2]. Additionally, we treated abuse of unspecific type as a separate category. To report the prevalence per type of abuse, we used percentages. Two reviewers clustered thematically associated factors of staff-to-resident abuse. They reported alongside the corresponding type of abuse. Additional information comprised the type of association (i.e., whether an associated factor of staff-to-resident abuse resulted from quantitative studies with statistically significant results [*p* < 0.05 or 0.01]), from quantitative studieswith statistically insignificant results (i.e., statistical significance was not available/applicable), or from qualitative studies. One reviewer extracted results on descriptions, experiences, and preventive interventions (second and third review question). After a topic-oriented categorisation, the reviewer tagged extracted data, paraphrased and finally thematically clustered data. This inductive approach facilitated the generation of themes directly from the extracted data [33].

## Results

**Fig. 1:**
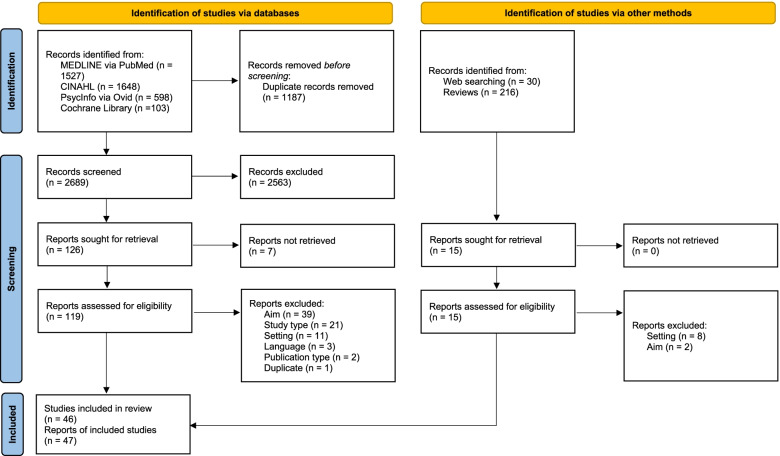
PRISMA 2020 flow diagram on the literature retrieval and selection process

Our search yielded 3876 references identified via databases and 246 references via supplementary search methods. After deduplication and title/abstract screening, we checked 141 full texts for eligibility. Finally, we included 46 studies reported in 47 references (one study was published in two separate reports) [34, 35] (Fig. 1).

Overall, the 47 study reports were published between 2000 and 2021, half of them since 2012 [9, 13, 36–57]. Most of the 46 studies were conducted in the USA (*n* = 9, 19.6%) [36, 38, 58–64], Norway (*n* = 8, 17.4%) [13, 42, 45, 46, 55, 56, 65, 66], and the UK (*n* = 7, 15.2%) [41, 47–49, 52, 53, 67]. The remaining studies took place in Israel (n = 4, 8.7%) [34, 35, 39, 68, 69], Canada (*n* = 3, 6.5%) [40, 70, 71], Taiwan (*n* = 3, 6.5%) [72–74], Czech Republic (*n* = 2, 4.3%) [75, 76], France (*n* = 2, 4.3%) [51, 57], and Switzerland (*n* = 1, 2.2%; this study addressed two of our review questions) [9, 50]. One study each (2.2%) was conducted in Germany [77], Korea [54], Portugal [44], Slovenia [37], Sweden [78], and Uruguay [43].

### Types, prevalence, and associated factors of staff-to-resident abuse

We identified 28 studies to answer our first review question on the types, prevalence, and associated factors of staff-to-resident abuse [9, 13, 34–38, 42, 44, 48–52, 54, 57–63, 66, 71–73, 75–77]. Detailed data on every study are available in supplementary material S2 and summarised in the following subsection.

Twenty-two out of the 28 studies (78.6%) had a cross-sectional design [13, 34–38, 42, 48, 49, 51, 52, 54, 57, 60–63, 66, 71–73, 76, 77]. Four studies (14.3%) were secondary data analyses [9, 44, 58, 59]. One study each (3.6%) was a qualitative study [50] and a qualitative study combined with a secondary data analysis [75]. Overall, 24,419 participants and/or documents from 1,432 nursing homes (data available for 19 out of 28 studies; median 24, IQR 12 to 105, range 5 to 369) were included (median 470, inter quartile range (IQR) 172 to 963, range 23 to 4,599). In the majority of studies, participants were nurses and nursing aides, followed by relatives or persons responsible for nursing home residents. Most of the studies used random sampling (*n* = 16, 57.1%) [9, 13, 34–38, 42, 54, 60–62, 66, 71–73, 76]. Five studies (17.9%) were based on a convenience sample [48, 51, 57, 63, 77], two studies (7.1%) on a complete sample [44, 58]. One study each (3.6%) used purposive [50] and snowball sampling [75]. Three studies (10.7%) did not report details on sampling [49, 52, 59]. All except one study (96.4%) used a retrospective reference time frame. This single study (3.6%) did not report details on reference time [54]. The reference time frame was mentioned in 20 studies (71.4%), varying between three months and unlimited, mostly one year (*n* = 11/20, 55%).

One study (3.6%) [44] reported sociodemographic and health-related abused residents. None of the studies mentioned staff-related characteristics.

Four out of 28 studies (14.3%) assessed a single type of abuse. Two of them investigated psychological abuse [72, 73], one study focussed on financial abuse [61], and one study on neglect [62]. Twenty-four studies (85.7%) assessed multiple types of abuse [9, 13, 34–38, 42, 44, 48–52, 54, 57–60, 63, 66, 71, 75–77], thereby covering two to eight types. Eighteen studies (64.3%) assessed psychological abuse [9, 13, 34–37, 42, 43, 48, 49, 52, 58–60, 71–73, 75–77]. Another 18 studies (64.3%) investigated physical abuse [9, 13, 34–38, 42–44, 48, 49, 52, 58–60, 71, 75–77]. Fifteen studies (53.4%) focussed on neglect [9, 13, 34, 35, 42–44, 48, 49, 52, 58–60, 71, 75–77], twelve studies (42.9%) on financial abuse [13, 34–37, 43, 48, 49, 58–60, 71, 75, 76], eleven studies (39.3%) on sexual abuse [13, 34–36, 38, 43, 48, 49, 58–60, 71, 76], and eight studies (32.1%) assessed other types of abuse [34–36, 43, 48, 49, 59, 60, 71, 75]. Six studies (21.4%) investigated abuse of unspecified type [50, 51, 54, 57, 63, 66].

For data collection, twenty-three out of 28 studies (82.1%) used a structured questionnaire [13, 34–38, 42, 48, 49, 51, 52, 54, 57, 59–63, 66, 71–73, 76, 77]. Two studies (7.1%) were based on multiple data collection methods. One of them used a structured questionnaire and a document analysis [9], the other one unstructured interviews and a document analysis [75]. One study each (3.6%) was based on structured interviews [50], document analysis [44], or did not report details on data collection methods [58]. Twenty-six studies (92.9%) used statistical methods for data analysis [9, 13, 34–38, 42, 44, 48, 49, 51, 52, 54, 57–63, 66, 71–73, 76, 77]. One study (3.6%) relied on qualitative phenomenological analysis and descriptive statistics [75], and one study (3.6%) used thematic analysis [50].

The prevalence of staff-to-resident abuse observed by staff was reported in nine out of 28 studies (32.1%) (Table 1; details on every study are available in supplementary material S2). The highest abuse rates observed by staff was related to abuse of unspecified type (51–76%) [13, 52, 76, 77] and lowest for sexual abuse (0–2%) [13, 36, 48, 76]. The prevalence of staff-to-resident abuse reported by abuse-committing staff was reported in eight out of the 28 studies (28.6%) (Table 1; details on every study are available in supplementary material S2). The highest abuse rates were committed by staff for inadequate care (87%) [65] and abuse of unspecified type (54–79%) [13, 34, 35, 52, 76, 77]. The prevalence of staff-to-resident abuse reported by nursing home residents and/or by others was reported in nine out of 28 studies (32.1%) (Table 1; details on every study are available in supplementary material S2). The highest abuse rates reported by residents and/or by others referred to psychological (4–99%) [37, 58–60, 72, 76] and physical abuse (< 1–93%) [37, 38, 44, 58–60, 76], as well as to neglect (16–87%) [44, 58–60, 62].

**Table 1:** Prevalence per type of staff-to-resident abuse (observed, committed, reported)

Type of abuse	Prevalence		
	Observed by staff	Committed/self-reported by staff	Reported by residents and/or others
Abuse of unspecified type ^+^	51–76% [13, 52, 76, 77]	54–79% [13, 34, 35, 52, 76, 77]	5–11% [76]
Psychological	20–62% [9, 13, 36, 48, 77]	30–46% [13, 42, 76]	4–99% [37, 58–60, 72, 76] ^§^
Physical	3–30% [9, 13, 36, 48, 76, 77]	6–40% [13, 42, 76, 77]	< 1–93% [37, 38, 44, 58–60, 76] ^§^
Sexual	0–2% [13, 36, 48, 76]	0–1% [13, 76]	6–40% [38, 58, 60] ^§^
Financial	0–25% [13, 36, 48, 61]	1–2% [13, 61]	6–72% [37, 58–60] ^§^
Neglect	9–58% [9, 13, 48, 76]	1–77% [13, 42, 76]	16–87% [44, 58–60, 62] ^§^
Other	4–91% [36, 65] ^*^	87% [65] ^**^	17–82% [59, 60] ^*** §^

*Note*s: ^+^ Including both observed and committed abuse for reference [52]

^*^ Other types of abuse including inadequate care, caregiving abuse, and medication abuse

^**^ Other types of abuse including inadequate care

^***^ Other types of abuse including caretaking abuse, mistreatment

^§^ Percentages correspond to the answers of participants having responded to items for reference [60]

Resident-related associated factors of staff-to-resident abuse were reported in twelve out of 28 studies (42.9%) (Table 2; details on every study are available in supplementary material S2). Staff-related factors of staff-to-resident abuse were mentioned in 16 of the 28 studies (57.1%) (Table 2; details on every study are available in supplementary material S2). Institution-related associated factors were reported in eleven of the 28 studies (39.3%) (Table 2; details on every study are available in supplementary material S2).

**Table 2:** Associated factors (resident-related, staff-related, and institution-related) per type of staff-to-resident abuse

Type of abuse	Associated factors (resident-related)	Non-associated factors (resident-related)
Abuse (no details on type of abuse)	Behaviour ^*^ [38, 71]	Health and functional status [38, 71]
	Health and functional status ^*^ [38, 54, 71]	Sociodemographic characteristics [38, 76]
	Other: resident-to-resident abuse ^*^ [38]	
	Sociodemographic characteristics ^*^ [38, 71]	
	Behaviour ^**^ [50]	
	Health and functional status ^**^ [50]	
	Sociodemographic characteristics ^**^ [50, 75]	
Psychological	Behaviour ^*^ [9, 42, 76]	Behaviour [9]
	Satisfaction with nursing home and care ^*^ [76]	Health and functional status [9, 34, 35]
		Satisfaction with nursing home and care [76]
		Sociodemographic characteristics [9, 34, 35]
Physical	Behaviour ^*^ [42, 76]	Health and functional status [34, 35, 44]
	Health and functional status ^*^ [76]	Sociodemographic characteristics [34, 35, 44]
	Sociodemographic characteristics ^*^ [34, 35, 76]	
Physical/psychological	Other: quality of life ^*^ [52]	Behaviour [52]
		Health and functional status [52]
Neglect	Behaviour ^*^ [9, 42]	Behaviour [9, 62]
	Health and functional status ^*^ [44, 62]	Health and functional status [9, 34, 35, 44, 62]
		Sociodemographic characteristics [9, 34, 35, 44, 62]
	**Associated factors (staff-related)**	**Non-associated factors (staff-related)**
Abuse (no details on type of abuse)	Attitude towards and experiences of abuse ^*^ [54]	Sociodemographic characteristics [54]
	Characteristics of personal life and personality ^*^ [57, 77]	Job-related characteristics [51]
	Coping strategies ^*^ [54, 77]	
	Emotional strain and burnout ^*^ [51, 57, 77]	
	Job-related characteristics ^*^ [51, 57, 73, 77]	
	Sociodemographic characteristics ^*^ [73]	
	Characteristics of personal life and personality ^**^ [75]	
	Coping strategies ^**^ [50]	
	Emotional strain and burnout ^**^ [75]	
	Job-related characteristics ^**^ [50]	
	Sociodemographic characteristics ^**^ [50]	
	Sociodemographic characteristics ^**^ [75]	
Psychological	Attitude towards and experiences of abuse ^*^ [34, 35]	Attitude towards and experiences of abuse [34, 35]
	Characteristics of personal life and personality ^*^ [76]	Characteristics of personal life and personality [34, 35, 42, 76]
	Coping strategies ^*^ [77]	Job-related characteristics [9, 34, 35, 42]
	Emotional strain and burnout ^*^ [9, 34, 35, 72, 76, 77]	Sociodemographic characteristics [9, 13, 34, 35, 42, 72, 76]
	Job-related characteristics ^*^ [9, 34, 35, 42, 72, 76]	
	Sociodemographic characteristics ^*^ [9, 13, 34, 35, 42, 72, 76]	
	Sociodemographic characteristics ^***^ [49]	
Physical	Attitude towards and experiences of abuse ^*^ [34, 35]	Attitude towards and experiences of abuse [34, 35]
	Characteristics of personal life and personality ^*^ [42, 76]	Characteristics of personal life and personality [34, 35, 76]
	Emotional strain and burnout ^*^ [34, 35]	Emotional strain and burnout [76]
	Job-related characteristics ^*^ [42, 76]	Job-related characteristics [34, 35, 42, 76]
	Sociodemographic characteristics ^*^ [13, 42]	Sociodemographic characteristics [13, 34, 35, 42, 49, 76]
	Sociodemographic characteristics ^***^[49]	
Physical/psychological	Emotional strain and burnout ^*^ [52]	
Sexual	Sociodemographic characteristics ^***^ [49]	
Financial	Attitude towards and experiences of abuse ^*^ [61]	
	Job-related characteristics ^*^ [61]	
	Sociodemographic characteristics ^***^ [49]	
Neglect	Attitude towards and experiences of abuse ^*^ [34, 35]	Attitude towards and experiences of abuse [34, 35]
	Characteristics of personal life and personality ^*^ [42]	Characteristics of personal life and personality [34, 35]
	Emotional strain and burnout ^*^ [34, 35, 51]	Emotional strain and burnout [9, 34, 35]
	Job-related characteristics ^*^ [9, 34, 35, 42, 51]	Job-related characteristics [9, 34, 35, 42, 51]
	Sociodemographic characteristics ^*^ [13]	Sociodemographic characteristics [9, 13, 34, 35, 42]
	Sociodemographic characteristics ^***^ [49]	
Other: Discriminatory abuse	Sociodemographic characteristics ^***^ [49]	
	**Associated factors (institution-related)**	**Non-associated factors (institution-related)**
Abuse (no details on type of abuse)	Facility characteristics ^*^ [54, 58, 63]	Facility characteristics [58, 63]
	Organization and culture of work ^*^ [54]	Organization and culture of work [58]
	Resident-related characteristics ^*^ [54]	Staff-related characteristics [54, 58]
	Staff-related characteristics ^*^ [58]	
	Organization and culture of work ^**^ [50, 75]	
	Staff-related characteristics ^**^ [50, 75]	
Psychological	Facility characteristics ^*^ [42]	Facility characteristics [9, 34, 35, 42, 72]
	Organization and culture of work ^*^ [9]	Organization and culture of work [9]
		Staff-related characteristics [9, 34, 35, 42]
Physical		Staff-related characteristics [34, 35, 42]
Physical/psychological		Facility characteristics [52]
		Staff-related characteristics [52]
Neglect	Facility characteristics ^*^ [34, 35, 42, 62]	Facility characteristics [9, 34, 35, 42]
	Organization and culture of work ^*^ [9]	Organization and culture of work [9]
	Resident-related characteristics ^*^ [62]	Staff-related characteristics [9, 34, 35]
	Staff-related characteristics ^*^ [34, 35]	Facility characteristics [34, 35, 42]

*Notes*: ^*^ Associated Factors in quantitative studies with statistically significant results (*p* > 0.05 or 0.01)

^**^ Associated factors in qualitative studies

^***^ Associated Factors in studies with statistically insignificant results (i.e., statistical significance was not available/applicable)

### Description and experience of staff-to-resident abuse

To answer our second review question on the description and experience of staff-to-resident abuse, we identified 14 studies [39–41, 43, 45, 53, 55, 56, 65, 67–70, 78]. Detailed data on every study are available in supplementary material S2 and summarised in the following subsection.

Ten out of 14 studies (71.4%) had a qualitative design [39–41, 43, 45, 53, 55, 56, 67, 70], three (21.4%) a cross-sectional design [65, 68, 69], and one study (7.1%) was a qualitative case study [78]. Overall, 1,250 participants were included (median 29, IQR 13 to 42, range 1 to 616) from 284 nursing homes (data available for eleven out of 14 studies; median 18, IQR 11 to 20, range 4 to 142). Mostly, nurses and nursing aides participated, followed by nursing home managers. The sampling strategy was predominantly purposive (*n* = 5, 35.7%) [39–41, 43, 67] or not reported (n = 4, 28.6%) [68–70, 78]. Three studies (21.4%) recruited on convenience [45, 53, 55] and one study each (7.1%) used a random [65] or theoretical sample [56]. In most of the studies, abuse was not specified (*n* = 10, 71.4%) [40, 41, 43, 53, 65, 67–70, 78]. Two studies (14.3%) addressed physical, psychological, financial, and sexual abuse as well as neglect [55, 56]. Neglect [39] or sexual abuse [45] were focussed in one study (7.1%). Interviews (of different types) were the most common data collection method (*n* = 11, 71.4%). Five of the interview-based studies used semi-structured interviews [39, 40, 53, 70, 78] and three studies relied on focus group interviews [41, 45, 56]. In one study, researchers used a structured interview [67]. One group of researchers combined focus group interviews with semi-structured interviews [55]. One study (7.1%) used a multi-methods approach combining interviews (not specified) and observations [43]. The three non-interview-based studies (21.4%) used a structured questionnaire [65, 68, 69]. For data analysis, three studies (21.4%) applied descriptive statistics [65, 68, 69]. Two of them additionally used regression analysis [68, 69]. Two studies each (14.3%) stated narrative analysis [53, 78], thematic analysis [40, 41], constant comparative analysis [55, 56] or did not specify the type of analysis [67, 70]. Two studies (14.3%) identified emerging themes without stating an explicit method [43, 45] and one study (7.1%) applied phenomenological analysis [39].

Five themes resulted from our analysis: (i) “Viewpoints on abuse”, (ii) “Tolerating abusive behaviours”, (iii) “Consequences and punishment”, (iv) “Reporting of abuse”, and (v) “Knowledge gaps”. (i) “Viewpoints on abuse” focused on abuse as a serious misconduct difficult to talk about. The conceptualization of abuse is often limited to physical abuse. Physical, sexual, and financial abuse are considered as most serious types of abuse. Abuse was described as contradicting leaders’ trust in their staff. Abuse might be intentional or unintentional. Harmful consequences of psychological abuse are not easy to identify. Sexual abuse could foster uncertainties of staff related to supporting residents with personal and intimate care. Abuse is often associated with negative feelings [40, 41, 43, 45, 55, 56, 70].

Two themes (ii and iii) reflect the ambiguity of participants confronted with abusive behaviour. (ii) “Tolerating abusive behaviours” was predicted by role conflicts, staff burnout, work stressors, and being married [68, 69]. (iii) “Consequences and punishment” referred to abuse as unacceptable practice requiring sanctions. Ridiculing a resident with dementia should result in advice and guidance of staff. Rough handling should entail verbal warning of staff. Stealing money requires dismissal and physical abuse should be reported to the police as a criminal offence [67]. (iv) “Reporting abuse” was associated with a potential for improvement.

Overall, participants characterized abuse as underreported. However, personal viewpoints of staff indicated that most carers are willing to anonymously report abuse. Based on the results of a nursing study cohort, the majority of participants stated that it is best to deal with abuse internally. Some participants would not report a resident’s abuse by a colleague since there are other ways to handle the situation. Other participants expressed fear to report abuse. They saw no use of reporting since nothing would change. Few participants did not feel brave enough to report abuse. Half of the participating nurses expected support from the management after reporting abuse. Two out of three stated that they would report abuse depending on its severity [41, 45, 56, 65]. (v) “Knowledge gaps” were related to a theoretical understanding of ‟neglect‟ and to staff education concerning sexual abuse [39, 45].

### Interventions aiming to prevent staff-to-resident abuse

To answer our third review question on preventive interventions, we identified five studies [36, 39, 45–47, 50, 64, 74]. Detailed data for every study are available in supplementary material S2 and summarised in the following subsection.

Four out of five studies (80.0%) had a qualitative design [46, 47, 50, 64] and one study (20.0%) was quasi-experimental using a before-after design [74]. Overall, 149 participants were included (data available for four out of five studies; median 19, IQR 14 to 43, range 12 to 100) from 42 nursing homes (data available for four out of five studies; median eight, IQR four to 15, range three to 23). Mostly, nurses and nursing aides were recruited, followed by nursing home managers. Two studies (40.0%) used purposive sampling [50, 64]. One study each (20.0%) used a convenience [46] or a random sample [47] or provided no details on sampling [74]. Predominantly, abuse of unspecified type (*n* = 3, 60.0%) [47, 50, 64]. One study each (20.0%) focussed on physical [46] or psychological abuse [74]. Interviews (of various types) were the most frequent method of data collection (*n* = 4, 80.0%) [46, 47, 50, 64]. Two interview-based studies used structured interviews [50, 64], one relied on study semi-structured interviews [47], and another one on focus groups [46]. One study used a structured questionnaire (one week before and one week after the intervention) [74]. Two studies (40.0%) used grounded theory methodology for data analysis [47, 64]. One study each (20.0%) applied descriptive statistics combined with inferential statistics [74], systematic text condensation [46], and thematic analysis [50].

The intervention study used a before-after design [74]. Nurses and nursing aides performed the intervention designed by the research team and a trained graduate nurse in the role of a facilitator. Every week, eight 90-min group-sessions took place with ten to twelve nurses. The sessions were part of a multi-component framework, including education and mutual support. The programme addressed aging-associated problems related to managing residents’ health problems, institutional elder abuse, factors associated with caregivers’ abusive behaviour, relaxation and stress management, dealing with a stressful caregiving context, and obtaining personal resources. Each session started with a 30-min lecture on the topic, followed by 40 min of informal exchange and mutual support among group members. The last 20 min were dedicated to an integrative discussion. Outcomes were caregivers’ psychological abusive behaviours, perceived level of work stress, and knowledge in geriatric care. Statistically significant pre-post effects comprised decreased psychological abusive behaviour on the part of nurses and improved knowledge about gerontology nursing. Self-rated level of work stress did not significantly decrease.

Concerning the four qualitative studies, our analysis yielded four themes on preventive strategies with regard to staff-to-resident abuse: (i) “Image of nursing”, (ii) “Organisational management”, (iii) “Organisational culture”, and (iv) “Skills and competencies”. (i) “Image of nursing” was related to staff recruitment. Improving the image of the nursing profession proved to be important to prevent abuse [50]. (ii) “Organisational management” covered recruitment of more and qualified staff, rotation, and management strategies ensuring rapid response to abuse [50]. (iii) “Organisational culture” focussed the encouragement of an open, supportive, reflective, and cooperative team culture facilitating to learn from each other (e.g., by fostering open-mindedness in discussing ethical dilemmas) (Q3, S. 1: 302) [46, 47, 50, 64]. (iv) “Skills and competencies” covered the importance of competencies, ongoing training, and education [46, 50, 64].

## Discussion

Our scoping review provides an overview of staff-to-resident abuse in nursing homes with regard to types of abuse, prevalence, associated factors, lived experience, and preventive interventions. The state of the current research comprises primarily cross-sectional studies, few secondary data analyses and qualitative studies, and only one quasi-experimental study addressing the prevention of staff-to-resident abuse. The prevalence rates reported in the studies varied widely, depending on the type of abuse. Associated factors were evaluated inconsistently. However, the results show factors influencing abuse at the staff, resident, and nursing home levels.

Our results yielded a wide range of prevalence estimates for all types of abuse. Possible reasons are different data collection methods but also the phenomenon as such. Staff-to-resident abuse is considered socially unacceptable [67]. Due to this, it is difficult to measure this phenomenon and to gain valid data. Firstly, different definitions of abuse may have guided the included studies, resulting in various operationalisations of abuse types in data collection instruments. The consequences are different variables, scales and accentuation of types of abuse. For example, willful ignoring is classified as neglect or physical or psychological abuse, depending on the study, which can lead to overlaps in prevalence surveys [79]. Secondly, the studies included different groups of people whose perceptions of abuse may vary. For example, “yelling” as a form of psychological abuse is determined by multiple factors and differs according to context and person [12]. Thirdly, reporting might be influenced by fear of consequences. Residents are dependent on nursing staff and, therefore, might be afraid of reporting abuse. Nursing staff members, on their part, are dependent on their employers and colleagues. Due to this, they also might refrain from reporting committed or observed abuse in order to protect others or themselves [65]. Furthermore, higher prevalence rates of observed abuse relative to committed abuse may indicate probable difficulties in admitting one’s own misconduct [13, 80]. Thus, it is crucial that data collection is non-judgemental and confidentiality in interviews is assured [81]. Fourthly, reporting abuse may be subject to recall bias by staff members (especially when reporting about observed abuse). For residents with cognitive impairment, reporting abuse is particularly difficult [81, 82]. In this context, different reference periods of data collection instruments may have influenced the prevalence rates. The large variation in prevalence data raises doubts about the validity of meta-analyses on this topic. Therefore, a cautious interpretation of the results is required [79]. However, prevalence data of the included studies show also substantial commonalities. The overall high prevalence rates of abuse of unspecified type (51–79%) [52, 77] indicate that abuse is not an uncommon phenomenon in nursing homes and should receive more attention. Multimorbid individuals with cognitive and/or functional limitations are frequent victims of abuse, regardless of the setting [83]. Staff members are in a position of power and control over mainly vulnerable and dependent persons – a fact contributing to the occurrence of abuse [3]. Psychological abuse is considered the most common type of staff-to-resident abuse and also the most common type of elder abuse in other settings [84]. Unlike in the non-community settings, the second most common type of abuse in nursing homes is physical abuse [3]. The high prevalence should receive special attention since the residents are highly vulnerable individuals who are dependent on staff. Their ability to defend themselves is limited due to cognitive and physical impairments [77, 85]. It is a matter of concern that physical abuse is the second most common type of abuse in non-community settings. Consistent with other studies [81, 84], sexual abuse proves to be the least common type reported. It is important to note that neglect maintains a special position. Unlike other types of abuse, it is perceived less as a personal misconduct than as a system misconduct. The changed perception of accountability may also change the reporting behaviour since staff members tend to find it easier to report behaviours that they do not consider to be their personal misconduct [80].

Staff-to-resident abuse is a multifactorial problem [35, 76, 86] determined by characteristics of the institution and the individuals involved (residents, staff members). However, we also found many contradictory results in the included studies. For numerous potential associated factors, a conclusive assessment is not possible. On the level of the residents, for example, various authors described functional status and certain aspects of health as risk factors for certain types of abuse [54, 71]. Not all authors confirmed this approach [35, 71].

On the staff level, we also identified inconsistent results in studies addressing the influence of job-related characteristics of abusive behavior. This refers to team relationships, work experience and workload [34, 50, 51, 76]. Also with regard to the influence of staff education, the studies show different results: while two found significant differences in terms of education level [13, 72], others found different results depending on the type of abuse or the source of expertise (e.g. geriatric training, academic or practical knowledge, college level education) [54, 76]. Still others found no significant differences in terms of education level [34, 35, 42]. Furthermore, our review also provides inconclusive results with regard to associated factors on the institutional level, including facility- and staff-related characteristics, for example, staff density, turnover, work organization and culture. In one study [54], job autonomy was statistically significantly associated with abuse. In another study [86], however, there was no statistically significant association of job autonomy with emotional abuse and neglect [86]. Possible reasons for these inconsistent findings may be study designs associated with various data collection methods in different settings as well as various study cohorts with different organizational cultures [11, 79, 84].

However, our review also yielded factors consistently associated with abuse or its subtypes. Among these factors are, for example, resident behavior with a focus on aggressive behaviour and neuropsychiatric symptoms of dementia [9, 42, 52, 76]. Staff members’ emotional strain and burnout were associated with several types of abuse [35, 52, 75]. Inadequate coping strategies, including not knowing personal limits or taking psychotropic medication to relieve stress, were significantly associated with some types of abuse [50, 54, 77]. Although our review does not provide conclusive results on all associated factors, we advocate for a comprehensive assessment of each context or situation. Associated factors contribute to residents’ vulnerability, thereby influencing their risk of being abused and their capacity to cope with abusive incidents [87]. Additionally, associated factors have an impact on staff members’ ability to balance demands and personal resources. Furthermore, associated factors have an effect on the increasing potential of abuse and may result from minor changes in the institutional system [88]. Therefore, it seems essential to conduct a comprehensive assessment in specific situations and to consider all possible associated factors.

Our results on the perception of abuse clearly reveal the ambiguity inherent in this topic. Although abusive behavior is considered “unthinkable” [56], it is often tolerated and underreported [65]. The findings also indicate the imbalance of power between residents not being able to draw attention on their experience of abuse, and staff members probably not reporting abuse in order to protect themselves or their colleagues. For the residents, however, abuse results in major consequences. Residents experience distress, long-term psychological consequences and physical injuries as well as lower quality of life and higher mortality [4]. Therefore, effective interventions aiming to prevent abuse in nursing homes are needed. Our results, however, reveal that research on preventive interventions is sparse. Only one interventional study [74] examined an intervention with components of education and mutual support in a quasi-experimental design. Four qualitative studies described the need for interventions addressing staff education and organizational conditions [46, 47, 50, 64]. Strategies promoting a critical reflection of situations and of one’s own behaviour should be embedded at the institutional level [46].

Nursing staff members are in the unique position to identify elder abuse in nursing homes. However, our results reveal that nurses also find themselves involved in ethical dilemmas without sufficient skills and resources to resolve them [12]. The imbalance between excessive demands and coping resources may increase the risk of abuse. To develop and to rigorously evaluate multidimensional approaches aiming to prevent abuse in nursing homes, more research is required. Interventions should be targeted at the individual and institutional level. Our results clearly reveal that system-wide associated factors of staff-to-resident abuse deserve particular attention. Future research can build on existing knowledge and initiatives on the community level, thereby focusing on the specifics of the nurse-resident-dyad [89]. To develop shared values and to facilitate authentic engagement, interventions may include public awareness campaigns, educational programs, supervision, as well as culture and practice development [12, 90]. Furthermore, a promising approach to intervention development may be a more comprehensive, less specified conceptualization of abuse, comprising resident-to-staff and resident-to-resident abuse as well. Interventions should aim at developing an organisational culture reflected in communication and behaviour that reduces the overall potential for abuse [79].

This scoping review has several strengths and limitations. The broad and comprehensive search strategy in databases, supplemented by a free web-search, yielded an extensive corpus of relevant international studies. However, we did not perform citation tracking to identify additional studies. Furthermore, our review does not include studies published before 2000 in languages other than English or German. It is restricted to publications in academic journals without considering, for example, grey literature. A strength of our procedure is the screening of studies performed by two researchers independently in order to minimize the risk of missing eligible studies. One researcher extracted data. To reduce errors, a second researcher confirmed data extractions. However, only one researcher analyzed and synthesized data. Although this is a limiting factor, it fostered an in-depth knowledge of the data. Other researchers of our group confirmed the results of the synthesis.

Since this is a scoping review, we did not assess the quality of the studies. Our presentation of the results does therefore not consider study quality. This limitation should be kept in mind when interpreting our data. Most of the included studies had a cross-sectional design. The heterogeneity of data collection entailed widely varying results. Facing this and the fact that we did not critically appraise the included studies, we cannot draw robust conclusions about the bias risk and its influence on the (true) prevalence of staff-to-resident abuse in nursing homes. Due to this, we cannot answer our research questions unequivocally. However, our scoping review provides an overview to readers to what is known about the prevalence and influencing factors of staff-to-residents abuse in nursing homes worldwide (with the limiting factors by using our design not considering the methodological quality of the individual studies). Additionally, our work could serve as a starting point for future primary research and systematic reviews considering methodological issues.

## Conclusions

This scoping review provides a comprehensive overview of what is known about staff-to-resident abuse in nursing homes. Data heterogeneity of the included studies does not allow concrete conclusions on prevalence data and definitive statements about the most significant factors influencing staff-to-resident abuse. Nevertheless, our results reveal that this type of abuse is a serious issue in institutional long-term care. The ambigious perception of staff-to-resident abuse as an unacceptable and still underreported behaviour point to characteristics of the dyad and their imbalance in power and vulnerability. Furthermore, it indicates a need for change in culture and conditions such as the establishment of a safe reporting culture and critical case reviews. Only few studies investigate preventive interventions. The limited amount of scientific literature implies the necessity for sound research in this field. However, existing knowledge provides guidance for relevant intervention components to prevent staff-to-resident abuse in nursing homes. In the future, researchers should develop multidimensional interventions including staff education as well as the development of organisational culture and conditions. Considering the serious consequences for the residents, particularly for highly vulnerable persons who are dependent on assistance, intervention development is acutely needed. Raising awareness and elaborating evidence-based guidance should have high priority in health care and research.

## Supplementary Information

**Additional file 1:** **Supplementary Material S1.** Search strategies per database

**Additional file 2:** **Supplementary Material S2.** Raw data per study

## Data Availability

Access to all data that was used in this study is open. See supplementary material S2 for the raw data set.
