# Supplementary material for: Staff-to-resident abuse in nursing homes: a scoping review
Source: BMC Geriatr. 2022 Jul 6;22:563. doi: 10.1186/s12877-022-03243-9 (PMC9261065; doi:10.1186/s12877-022-03243-9)
Supplement: Supplementary file 1 — Additional file 1: Supplementary Material S1. Search strategies per database [file 12877_2022_3243_MOESM1_ESM.pdf]

Staff-to-resident abuse in nursing homes: a scoping review

Supplementary material
Electronic database search strategies

Date: 23.06.2020

MEDLINE via PubMed

#	String	Hits
**1**	VIOLENCE[TIAB] OR ABUSE[TIAB] OR MISTREATMENT[TIAB] OR MALTREATMENT[TIAB] OR NEGLECT[TIAB] OR AGGRESSION[TIAB] OR ASSAULT[TIAB] OR "INADEQUATE CARE"[TIAB] OR NEGLECTION[TIAB] OR DISREGARD*[TIAB] OR NEGLIGENCE[TIAB] OR "ELDER ABUSE"[MESH] OR "PHYSICAL ABUSE"[MESH] OR AGGRESSION[MESH]	241754
**2**	"LONG-TERM CARE"[TIAB] OR "LONG TERM CARE"[TIAB] OR "NURSING HOME*"[TIAB] OR "CARE HOME*"[TIAB] OR "RESIDENTIAL HOME*"[TIAB] OR "RESIDENTIAL CARE HOME*"[TIAB] OR "RESIDENTIAL FACILIT*"[TIAB] OR "INSTITUTIONAL CARE"[TIAB] OR "INSTITUTIONALISED CARE"[TIAB] OR "INSTITUTIONALIZED CARE"[TIAB] OR "HOMES FOR THE AGED"[MESH] OR "NURSING HOMES"[MESH] OR "LONG-TERM CARE"[MESH]	87548
**3**	(VIOLENCE[TIAB] OR ABUSE[TIAB] OR MISTREATMENT[TIAB] OR MALTREATMENT[TIAB] OR NEGLECT[TIAB] OR AGGRESSION[TIAB] OR ASSAULT[TIAB] OR "INADEQUATE CARE"[TIAB] OR NEGLECTION[TIAB] OR DISREGARD*[TIAB] OR NEGLIGENCE[TIAB] OR "ELDER ABUSE"[MESH] OR "PHYSICAL ABUSE"[MESH] OR AGGRESSION[MESH]) AND ("LONG-TERM CARE"[TIAB] OR "LONG TERM CARE"[TIAB] OR "NURSING HOME*"[TIAB] OR "CARE HOME*"[TIAB] OR "RESIDENTIAL HOME*"[TIAB] OR "RESIDENTIAL CARE HOME*"[TIAB] OR "RESIDENTIAL FACILIT*"[TIAB] OR "INSTITUTIONAL CARE"[TIAB] OR "INSTITUTIONALISED CARE"[TIAB] OR "INSTITUTIONALIZED CARE"[TIAB] OR "HOMES FOR THE AGED"[MESH] OR "NURSING HOMES"[MESH] OR "LONG-TERM CARE"[MESH])	2082
**4**	#4 AND FILTERS: FROM 2000 - 2020	1527

CINAHL via EBSCO

#	String	Hits
**1**	TI VIOLENCE OR TI ABUSE OR TI MISTREATMENT OR TI MALTREATMENT OR TI NEGLECT OR TI AGGRESSION OR TI ASSAULT OR TI "INADEQUATE CARE" OR TI NEGLECTION OR TI DISREGARD* OR TI NEGLIGENCE OR AB VIOLENCE OR AB ABUSE OR AB MISTREATMENT OR AB MALTREATMENT OR AB NEGLECT OR AB AGGRESSION OR AB ASSAULT OR AB "INADEQUATE CARE" OR AB NEGLECTION OR AB DISREGARD* OR AB NEGLIGENCE OR MH "ELDER ABUSE"	118762
**2**	TI "LONG-TERM CARE" OR TI "LONG TERM CARE" OR TI "NURSING HOME*" OR TI "CARE HOME*" OR TI "RESIDENTIAL HOME*" OR TI "RESIDENTIAL CARE HOME*" OR TI "RESIDENTIAL FACILIT*" OR TI "INSTITUTIONAL CARE" OR TI "INSTITUTIONALISED CARE" OR TI "INSTITUTIONALIZED CARE" OR AB "LONG-TERM CARE" OR AB "LONG TERM CARE" OR AB "NURSING HOME*" OR AB "CARE HOME*" OR AB "RESIDENTIAL HOME*" OR AB "RESIDENTIAL CARE HOME*" OR AB "RESIDENTIAL FACILIT*" OR AB "INSTITUTIONAL CARE" OR AB "INSTITUTIONALISED CARE" OR AB "INSTITUTIONALIZED CARE" OR MH "NURSING HOMES" OR MH "LONG TERM CARE"	69469
**3**	#1 AND #2	1843
**4**	#3 AND LIMITERS - PUBLISHED DATE: 20000101-20201231	1647

PsycINFO via OVID

#	String	Hits
**1**	(VIOLENCE OR ABUSE OR MISTREATMENT OR MALTREATMENT OR NEGLECT OR AGGRESSION OR ASSAULT OR "INADEQUATE CARE" OR NEGLECTION OR DISREGARD* OR NEGLIGENCE).TI,AB. OR EXP "ELDER ABUSE"/	239816
**2**	("LONG-TERM CARE" OR "LONG TERM CARE" OR "NURSING HOME*" OR "CARE HOME*" OR "RESIDENTIAL HOME*" OR "RESIDENTIAL CARE HOME*" OR "RESIDENTIAL FACILIT*" OR "INSTITUTIONAL CARE" OR "INSTITUTIONALISED CARE" OR "INSTITUTIONALIZED CARE").TI,AB. OR EXP "NURSING HOMES"/ OR EXP "LONG TERM CARE"/	23714
**3**	#1 AND #2	1304
**4**	LIMIT #3 TO YR="2010 -CURRENT"	598

Cochrane Library

#	String	Hits
**1**	VIOLENCE:TI,AB OR ABUSE:TI,AB OR MISTREATMENT:TI,AB OR MALTREATMENT:TI,AB OR NEGLECT:TI,AB OR AGGRESSION:TI,AB OR ASSAULT:TI,AB OR "INADEQUATE CARE":TI,AB OR NEGLECTION:TI,AB OR DISREGARD*:TI,AB OR NEGLIGENCE:TI,AB OR MH "ELDER ABUSE" OR MH "PHYSICAL ABUSE" OR MH AGGRESSION	14618
**2**	"LONG-TERM CARE":TI,AB OR "LONG TERM CARE":TI,AB OR "NURSING HOME*":TI,AB OR "CARE HOME*":TI,AB OR "RESIDENTIAL HOME*":TI,AB OR "RESIDENTIAL CARE HOME*":TI,AB OR "RESIDENTIAL FACILIT*":TI,AB OR "INSTITUTIONAL CARE":TI,AB OR "INSTITUTIONALISED CARE":TI,AB OR "INSTITUTIONALIZED CARE":TI,AB OR MH "HOMES FOR THE AGED" OR MH "NURSING HOMES" OR MH "LONG-TERM CARE"	4955
**3**	#1 AND #2	139
**4**	#3 WITH PUBLICATION YEAR FROM 2000 TO 2020	103
